# Robotically Controlled Terahertz Probe for In Vivo Skin Evaluation: Imaging with the PicoBot

**DOI:** 10.1007/s10762-025-01055-7

**Published:** 2025-05-30

**Authors:** Jacob J. Young, Agrima Agarwal, Benjamin G. Page, Anubhav Dogra, Arturo I. Hernandez-Serrano, Joseph Hardwicke, Emma Pickwell-MacPherson

**Affiliations:** 1 Physics, University of Warwick, Gibbet Hill Campus, CV4 7AL Coventry, Warwickshire UK; 2 Institute of Applied & Translational Technologies in Surgery (IATTS), University Hospital Coventry and Warwickshire, Coventry, CV2 2DX Warwickshire UK

**Keywords:** Terahertz, Skin evaluation, Skin cancer, Imaging, In vivo

## Abstract

In this work, we demonstrate significant modifications to our robotically controlled terahertz (THz) sensing system, the “PicoBot,” enabling it to perform in vivo imaging of skin rather than limiting it to single-point measurements. By integrating a robotic arm equipped with force-sensitive feedback control, we maintain consistent contact pressure between the probe and the skin surface throughout imaging. In conjunction with this hardware advancement, we introduce an accompanying image analysis pipeline that reduces noise and enhances repeatability across scans. These improvements allow for reliable intra- and inter-subject comparisons, a critical step toward the clinical utility of THz imaging. Our ultimate aim is to use THz imaging to detect skin cancer margins: this paper highlights progress towards this goal and skin evaluation in general.

## Introduction

In this work, we present the second generation of our robotically controlled terahertz (THz) device, called the “PicoBot”. Originally only able to make single-point measurements [1], the PicoBot is now able to take hyperspectral THz images of skin in vivo. Due to the sensitivity of THz radiation to subtle changes in skin properties, our aim is to develop the PicoBot for use as a skin evaluation tool that could be used by clinicians for the diagnosis and monitoring of skin conditions including skin cancer.

Several research groups have investigated the use of THz sensing as a diagnostic tool for various diseases. For example, tissues with cancer typically have greater hydration than surrounding tissue due to increased vascularity, providing a contrast in time-domain transmission spectroscopy of ex vivo samples, as shown in a study of breast cancer by Ashworth et al. [2]. In 2011, Reid et al. demonstrated a distinct difference in the THz response of ex vivo diseased and healthy colon tissue [3]. Following these successful studies, techniques, and technology have been developed for a range of in vivo applications, such as Hernandez-Cardos’ demonstration of THz sensing for diabetic foot syndrome [4], and Lamberg et al.’s technique for cornea imaging [5]. Each of these studies showed a contrast between the THz response of healthy and diseased tissue results, but the small sample sizes used in the studies meant that further in vivo measurements are needed to understand contrast mechanisms.

Hernandez-Serrano et al. developed a portable handheld THz device for in vivo skin sensing [6]. This device, used with the skin measurement protocol by Lindley-Hatcher et al. [7], measured 300 volunteers. Skin parameters for these volunteers were calculated using the stratified medium theory model [8] and compared to survey responses by Ding et al. [9] to identify participant-invariant parameters. Ding also used the handheld device to evaluate the effects of transdermal moisturising patches on the skin in order to determine the efficacy of different backing materials. These studies have shown a sensitivity of THz to the different hydration states of the skin caused by lifestyle factors and the application of moisturising patches, further increasing the interest in the use of THz as a skin evaluation modality.

Harris et al. [10] developed a handheld imaging probe called the PHASR. It can capture a 12 mm by 19 mm image with 1 mm resolution in approximately 70 s. This device uses a photo-conductive antenna, providing phase information. To achieve this speed, it employs an asynchronous optical sampling system (ASOPS) instead of the more common mechanical optical delay unit (ODU). The ODU has a much lower sampling rate of about 10Hz, compared to 100Hz for the ASOPS. The speed of this acquisition is suitable for in vivo THz imaging, and the group led by Arbab is investigating how it could be used to grade the severity of burn wounds [11].

A recent study by the MacPherson THz group was the first study using THz radiation to examine skin cancer and dry skin conditions of patients in vivo in a hospital setting. The study compared eczema, psoriasis, and healthy skin, and the effects of moisturiser on these conditions. A handheld probe capable of taking a point measurement in the region of interest was used in this study. The results showed that these three conditions could be distinguished by their THz response and how the moisturiser affected the THz response as it soaked in [12]. When comparing skin cancers to healthy skin, a clear difference was observed, but measurements of single points on different patients in different locations on the body made it difficult to observe statistically significant trends [13]. The study concluded that an imaging scheme is needed to better compare diseased skin regions to the surrounding healthy skin.

The refractive index of skin at 1 THz is close to 2. Non-contact imaging would therefore cause large losses from the impedance mismatch between air and skin. Additionally, non-contact imaging would make it harder to get the sample in focus. We therefore have developed a contact imaging approach to optimise signal strength, alignment, and stability. However, this means that a further challenge with THz in vivo skin imaging is maintaining consistent pressure on the sample. It has been shown by Wang et al. that the THz response of the skin depends on the applied pressure on the skin [14], and this effect is usually mitigated by a pressure sensor and the careful operation of the user [7, 12, 13, 15]. However, this is only as reliable as the user and reduces the repeatability of the measurements. We have therefore incorporated our THz probe with a robotic arm, to develop the PicoBot [1]. Employing protocols laid out in [1], the PicoBot uses a KUKA medical-grade robotic arm (LBR-Med) to ensure stable, repeatable measurements and mitigate the pressure sensitivity of the THz response of the skin. In this way, the PicoBot can take point measurements of different locations on the body with reliable pressure control.

In this paper, the capability of the PicoBot is extended to image an area of skin rather than only make a simple point measurement. This is non-trivial as the contact pressure must be maintained across the image area throughout the duration of the measurement and also be comfortable for the patient. The scanning is therefore done by incorporating a motorised *XY* stage inside the probe head. This moves the THz beam across the imaging window, enabling the THz probe to be positioned in one place on the patient throughout the measurement. This also avoids potential problems, such as knocking scabs off that could be caused if the probe were to be dragged across the skin. By considering the numerous variables and parameters involved, we also present a noise-mitigation data processing technique which significantly improves the comparative analysis capabilities. The new PicoBot capabilities are already being used in our latest in vivo study of skin cancer patients at UHCW. The following sections introduce the instrumentation and analysis techniques we have developed.

## THz Skin Scanning Instrumentation Development

### The PicoBot

The PicoBot is a THz probe integrated into a robotic arm such that the probe can be positioned on the skin with constant pressure. One of the key design considerations was the clinical environment the probe needs to function in, this required imaging speed to be balanced with a robustness and ease of maintenance. Further details of the robotic control and calibration are given in reference [1].

The probe is positioned by a KUKA robotic arm at a location on the skin identified using computer vision techniques. Once in contact with the skin, the probe maintains constant pressure. This protocol has been published by Dogra et al. in [1]. The probe is named the PicoBot due to its robotic movement and the picosecond measurement time scale in the THz regime.

In this work, we have incorporated an *XY* translation stage so as to be able to image an area of skin, rather than just take a point measurement. The scanning trajectory has been carefully designed so as to minimise the acquisition time as much as possible whilst maintaining as much image detail as feasible. Additionally, we have found that as well as removing water vapour from the air, purging the probe head with dry air or nitrogen improves the signal-to-noise ratio considerably. It is worth noting that the signal-to-noise ratio in newer THz systems such as the Menlo TeraSmart is significantly better than in older systems previously used by the MacPherson group and there is no need to do the baseline subtraction [15] done in previous THz analysis regardless of purging. With these new imaging capabilities in the probe head, we have also revised the data processing.

#### PicoBot Probe Head Design

The PicoBot probe head contains a Thorlabs M30XY *XY* motorised translation stage that raster scans a Menlo THz reflection probe, allowing its focused THz beam to move across a quartz imaging window. This was chosen over a galvo scanner (such as that used in [10]) because it simplifies the focusing optics and allows the use of the Menlo reflection head, which is a robust easy to maintain system, particularly suited to a clinical environment. The stage and reflection probe are mounted inside a 3D-printed casing that fixes the optical path length between the reflection head and the imaging window. Shims have been included so that the path length can be altered without introducing instability into the design. The casing has a hole for wires and fibre optic cables to pass into the device and a second hole to allow a dry air feed to purge water vapour from the system. This reduces the attenuation of the THz in the air and improves the signal-to-noise ratio. A mounting bracket has been included for an endoscopic camera to be incorporated into the probe, allowing the sample to be photographed through the imaging window.

**Fig. 1:**
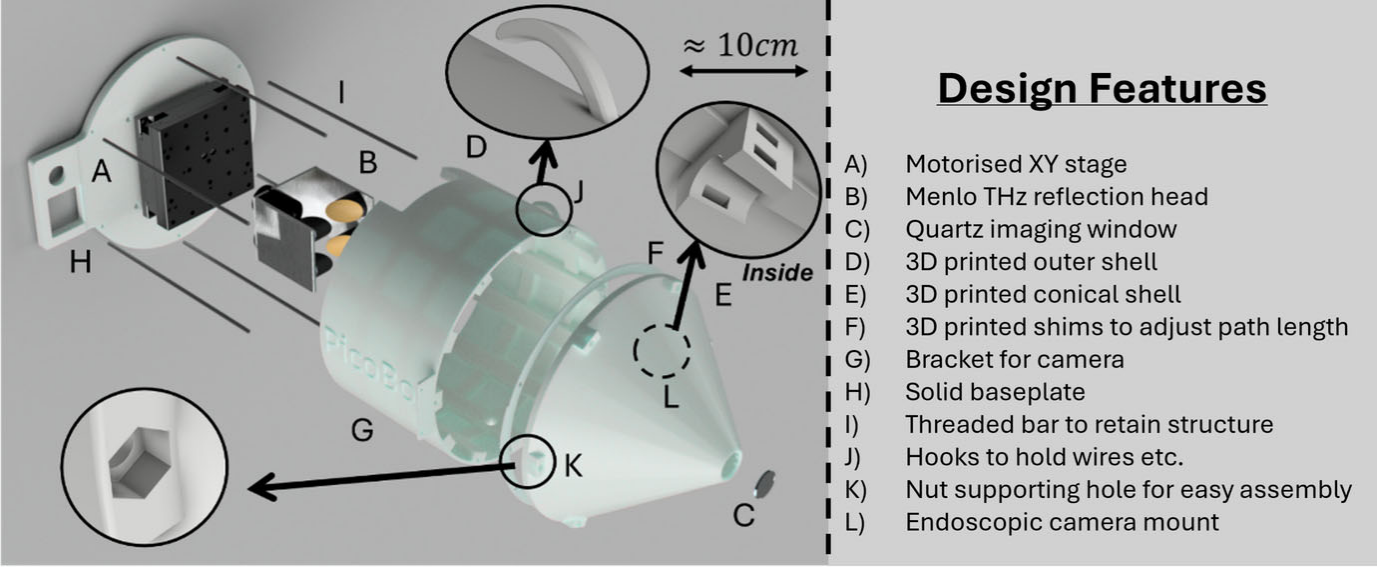
Construction of the PicoBot imaging probe

Figure 1 shows an exploded view of the PicoBot’s construction.

**Fig. 2:**
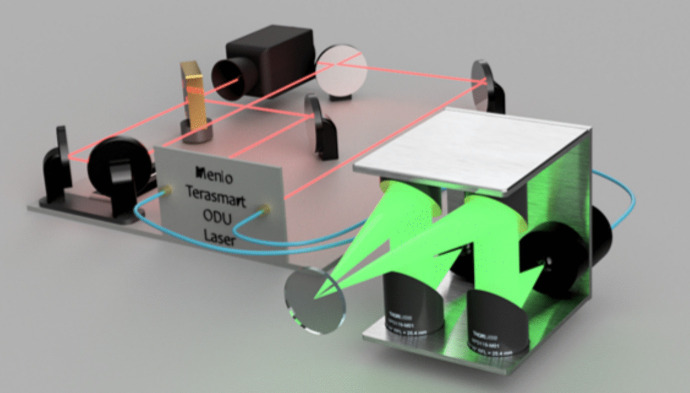
A diagram showing how the Menlo Terasmart is incorporated into the PicoBot probe

A diagram showing the working of the THz reflection set up used in the PicoBot is shown in Fig. 2: a femtosecond laser is used to excite an InGaAs photoconductive emitter, and the THz beam is then focused by a set of parabolic mirrors onto a quartz imaging window with an incident angle of $8.8^\circ$. The inclusion of an imaging window allows alignment to be maintained with the sample. The THz reflection from the sample is focused onto a photoconductive detector by a second pair of parabolic mirrors. The photoconductive detector is pumped by a probe beam from the femtosecond laser, with an arrival time controlled by an optical delay unit (ODU).

**Fig. 3:**
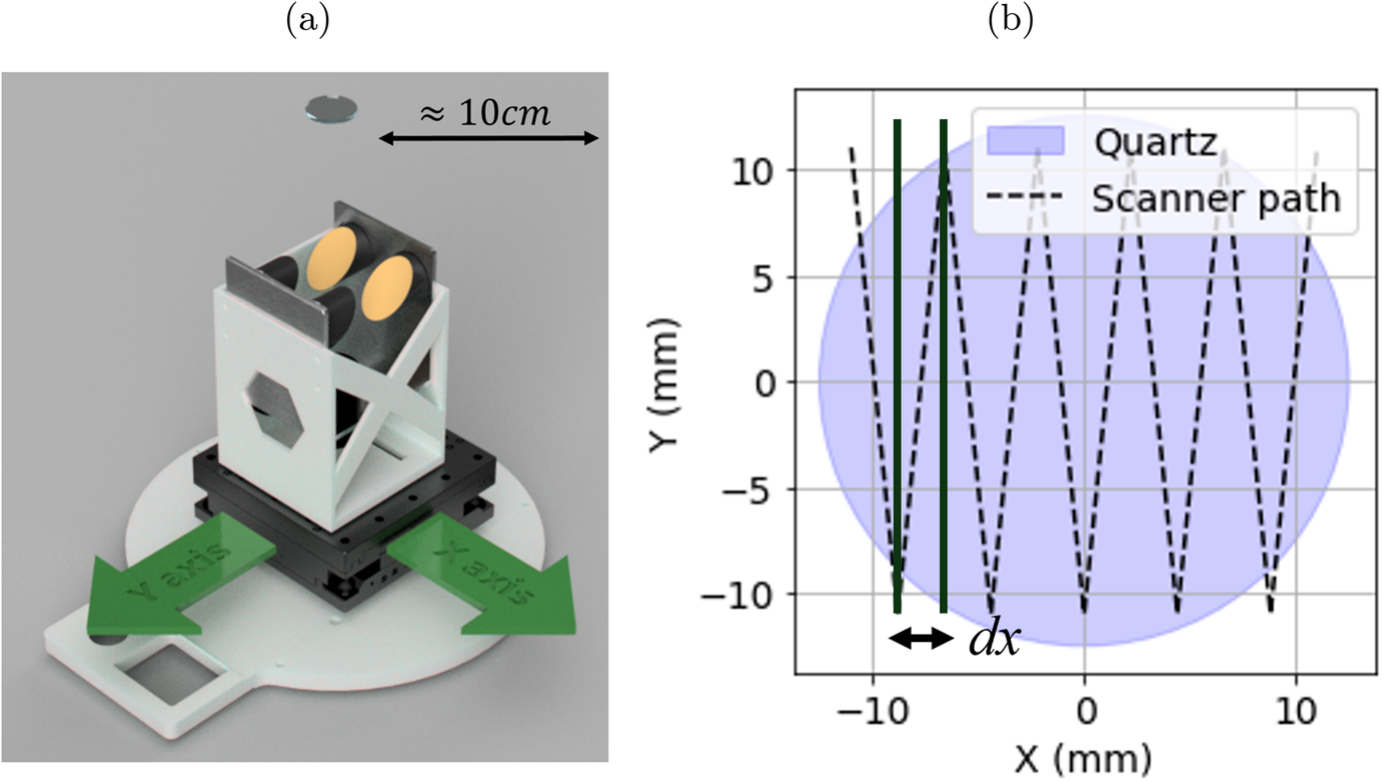
**a** The *XY* stage, the Menlo THz reflection head and quartz window with the *X* and *Y* axes shown in green lines. **b** Raster scan path used to acquire an image. The stage moves in a continuous motion without stopping

### Raster Scan Path

The raster scan is performed with continuous movement in a zigzag pattern, without stopping at each coordinate. Figure 3a shows a diagram illustrating the movement axes of the *XY* stage and the reflection head in relation to the quartz imaging window, and the movement path is shown in Fig. 3b. Image size is limited to 25 mm diameter by the quartz imaging window although the *XY* stage has a range of up to 30 mm.

As the probe moves, THz measurements are made continuously, while the stage reports the position immediately before and after each measurement. Approximately 100 waveforms are measured in each single line. The resolution is determined by both the diffraction-limited spot size of the THz beam (typically $\approx 5mm$) and the line spacing in the raster path, labelled *dX* in Fig. 3b. Usually, $dX = 1mm$ is chosen. This scan path is chosen as the Thorlabs M30XY stage requires the stage to stop at each way point rather than having the functionality to set waypoints that are moved through continuously. Due to latency issues in the translation stage, some of the coordinates are incorrectly assigned and so several *ms* of sleep time were added each time the stage reaches a waypoint. In the discussion section, possible improvements that involve the acquisition of a different *XY* stage will be discussed; however, the Thorlabs M30XY stage has been chosen at present as it is compact, light, affordable, and has a position repeatability of 2.5 $\mu m$ that is well beyond resolution limits imposed by the diffraction-limited spot size of the THz beam.

### Imaging Protocol

**Fig. 4:**
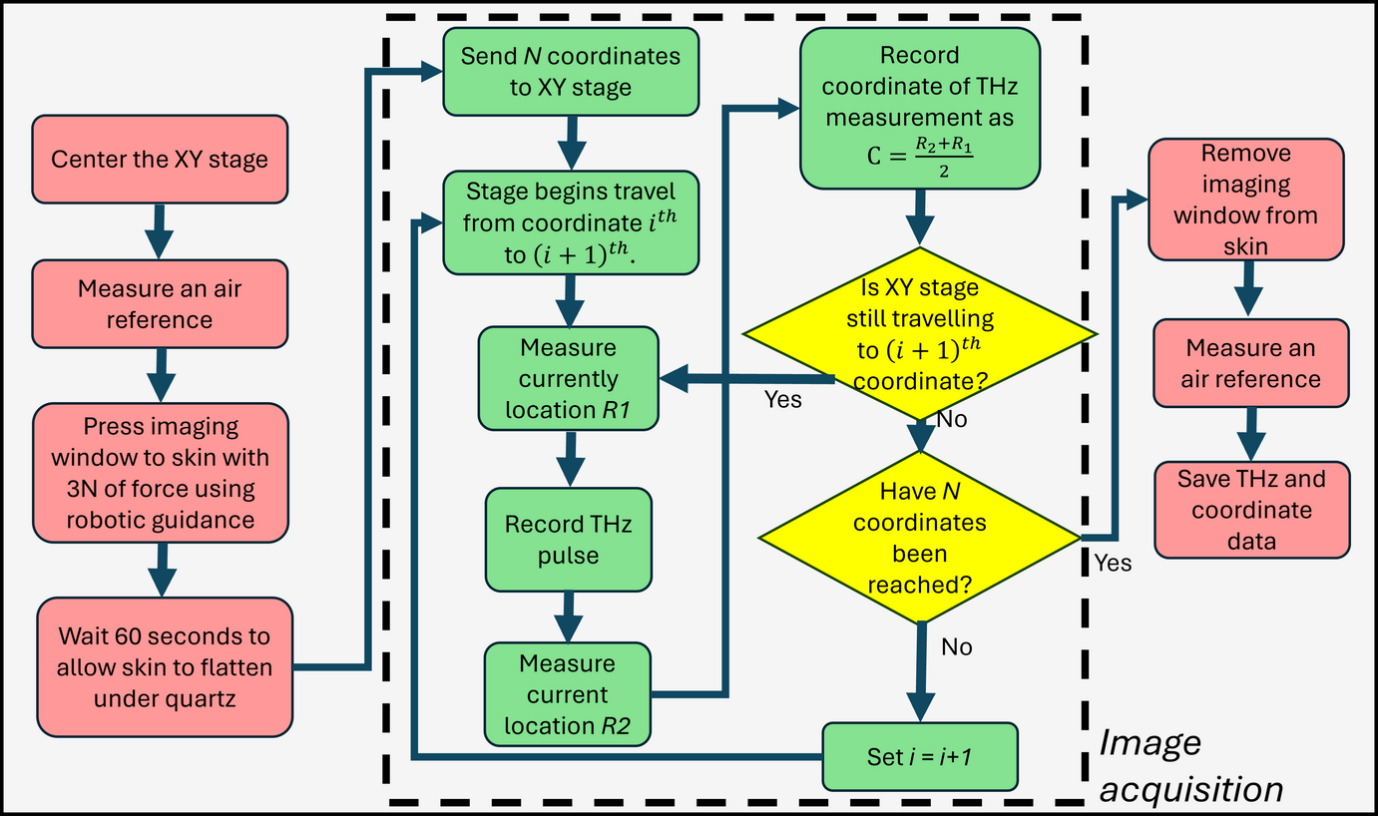
Flowchart of the PicoBot imaging protocol for an in vivo measurement of skin. The image acquisition process itself is outlined with a dashed box

**Fig. 5:**
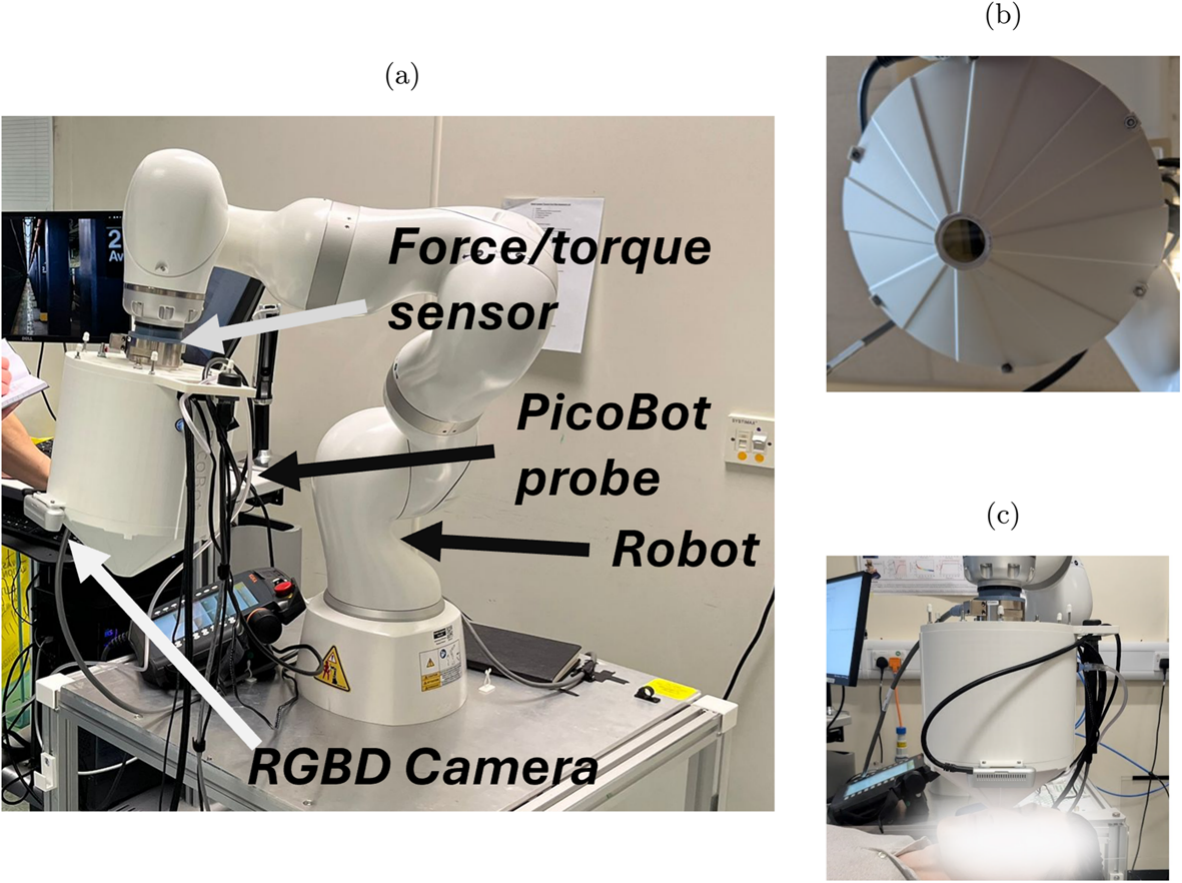
Side profile of the PicoBot probe. The base is attached to a robotic arm via a force sensor, this ensures accurate guidance of the PicoBot to the relevant area of skin, and consistent application of a mild force so the quartz imaging window makes good contact with the skin. The 3D camera used to guide the PicoBot can also be seen in the photo. The quartz imaging window of the PicoBot probe. The PicoBot imaging the right cheek of a volunteer

When imaging people, there are many variables to consider. In particular, it is important to be aware that pressing the imaging window on the skin affects the reflected THz signal depending on the contact pressure and duration of contact. The change is greatest in the first 60 s and seems to be mostly due to flattening the skin [13]. In our protocol, the PicoBot is held in contact with the skin with 3N force and this is done for 60 s before starting image acquisition. The robotic arm provides consistent contact between the imaging window and the skin and is much more repeatable than using a handheld device [1]. The imaging process for in vivo measurements is explained in the flowchart in Fig. 4. Photos of the fully realised PicoBot probe are shown in Fig. 5. A 2.5 cm × 2.5 cm area measurement can be performed in 4 min, and with an additional minute to allow the skin to adjust to the probe pressure: it takes a total of 5 min for each area of skin imaged.

## Development of Signal Processing

To characterise a sample across the 2.5 cm diameter image, the quartz must be uniform (or its thickness and refractive index characterised as a function of position), and the THz spectrum emitted by the photoconductive antenna should ideally remain stable throughout the 4-min measurement time. Self-referencing methods similar to those in references [4, 16] are used to account for fluctuations in the incident THz signal with time and also position. For in vivo measurements, the sample is living and can move and/or change with time, creating an added complication. In this section, we therefore carefully explain how to isolate the relevant THz signals so as to create meaningful and repeatable analysis during skin imaging.

**Fig. 6:**
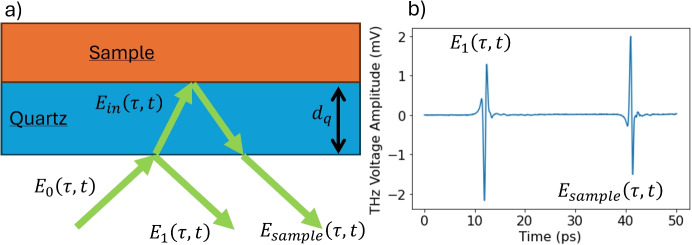
**a** Example of the THz beam path through a quartz window reflected from a quartz-sample boundary. **b** The measured THz waveform consists of 2 pulses, $E_{1}(\tau , t)$ and $E_{sample}(\tau , t)$ and are shown in the time domain

Figure 6 shows the THz path through the quartz window to the sample boundary and back through the quartz. The reflection from the air-quartz interface produces the first pulse ($E_1 (\tau , t)$) in the time-domain waveform, while the reflection from the quartz sample manifests as the second pulse ($E_{sample}(\tau , t)$). $\tau$ is the time the measurement is made at, and *t* is the optical delay between the emission of the THz radiation and the detection of the THz field, measured in picoseconds. The pulse is broadband, so each reflection contains a range of frequency ($\omega$) values.

### Temporal Stability

**Fig. 7:**
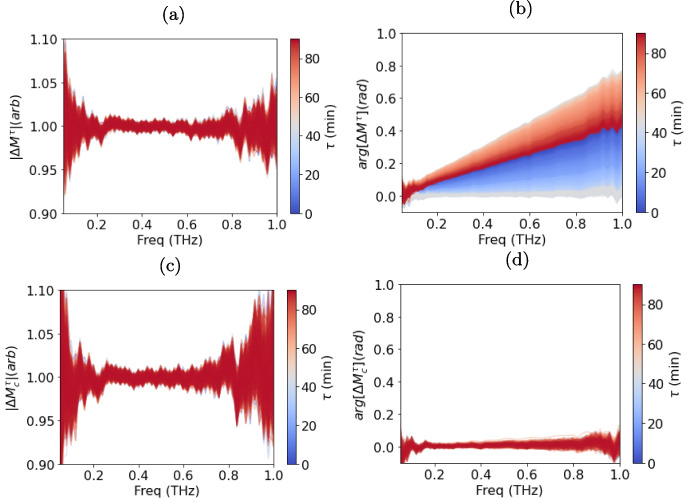
The change in the **a** amplitude and **b** phase of a measurement of air at a signal point over 90 min, with measurements made at a rate of 10Hz. **c** The change in amplitude and **d** of the corrected measurements of air made over 90 min, calculated using Eq. 8

The $\tau$ dependence of $E_{sample}(\tau , t)$ was determined by measuring air at a single point on the quartz window for 90 min and examining the relative change in the phase and amplitude of the signal:

1 $$\begin{aligned} \Delta M^{\tau }(\tau , \omega ) = \frac{\mathcal {F}\{E_{sample}(\tau , t)\}}{\mathcal {F}\{E_{sample}(90 \text { min}, t)\}}, \end{aligned}$$

where $\mathcal {F}$ is the Fourier transform operator. Figure 7a shows the amplitude $|\Delta M^{\tau }(\tau , \omega )|$, and Fig. 7b shows the phase $\arg [\Delta M^{\tau }(\tau , \omega )]$ throughout the 90-min measurement of air. The amplitude exhibits approximately $5\%$ noise at high and low frequencies, while the phase systematically varies throughout the measurement. This suggests that $\arg [\Delta M^{\tau }(\tau , \omega )]$ is $\tau$-dependent over the time scales of a measurement. This variation could be caused by fibre drift or thermal effects in the Menlo optical delay unit.

### Spatial Stability

**Fig. 8:**
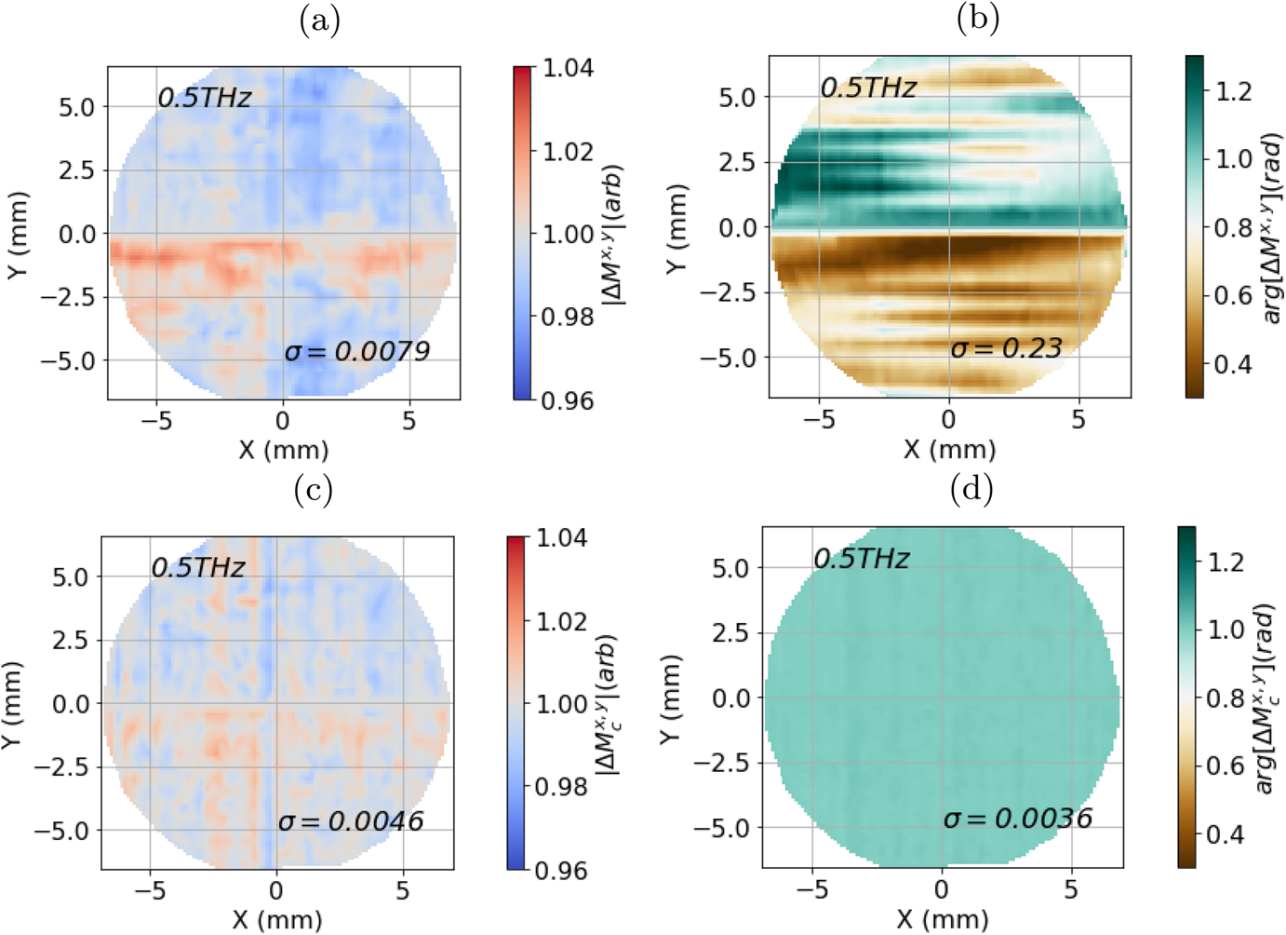
The change in the **a** amplitude and **b** phase at 0.5THz of an image of air taken with PicoBot. **c** The change in amplitude and **d** of the image formed of the corrected measurements of air, calculated using Eq. 9. The standard deviation for each of the parameters plotted is given by $\sigma$

During the raster scan, the movement of the fibres and any inhomogeneity in the quartz window can cause variations in the measurements. Therefore, it is important to quantify the spatial stability of the measurement as the raster scan is carried out. An image of air has been measured for this reason. The change in the measurement across the image can be quantified using:

2 $$\begin{aligned} \Delta M^{x,y}(\tau ,x,y,\omega ) = \frac{\mathcal {F}\{E_{sample}(\tau ,x,y,t)\}}{\mathcal {F}\{E_{sample}(\tau _{0},X=0,Y=0,\omega )\}}, \end{aligned}$$

where $\tau _{0}$ is the time when the pixel at $X=0$ and $Y=0$ was measured. The amplitude $|\Delta M^{x,y}(\tau ,x,y,\omega =0.5 \text {THz})|$ is plotted in Fig. 8a, and the phase $\arg [\Delta M^{x,y}(\tau ,x,y,\omega =0.5 \text {THz})]$ is plotted in Fig. 8b. The amplitude varies between 0.97 and 1.03 across the image, while the phase varies between 0.3 and 1.3 radians. The variation in both cases is systematic: repeating the measurement several times yields very similar results. Figure 7b shows how the phase changes with time over a 90 min measurement. Since the image of air takes over 4 min to acquire, the effects of the phase instability can be seen in Fig. 8b.

### Correcting Temporal and Spatial Instability

Both the temporal ($\tau$) and positional (*X*, *Y*) dependence of the THz signal can be accounted for by using the initial reflection from the air-quartz boundary to characterise the incident THz signal for that measurement.

It is useful to relate the THz electric field incident on the sample ($E_{in}(\tau , t)$) to the field incident on the air-quartz boundary ($E_0(\tau , t)$):

3 $$\begin{aligned} \mathcal {F}\{E_{in}(\tau , t)\} = \mathcal {F}\{E_0(\tau , t)\} T_1(x,y,\omega ), \end{aligned}$$

where $T_1(x,y,\omega ) = t_{aq}(\omega ) e^{-\alpha _q(\omega ) d_q(x,y)} e^{-j\frac{\omega }{c}n_q(\omega )d_q(x,y)}$ is a field modification parameter corresponding to transmission through the air-quartz boundary and passing through the quartz to the sample boundary. $t_{aq}(\omega )$ is the transmission coefficient at the air-quartz boundary, $\alpha _q(\omega )$ is the extinction coefficient in quartz, $d_q(x,y)$ is the thickness of the quartz as a function of position, and $n_q(\omega )$ is the refractive index of quartz. As the beam is focused on the outer quartz to air boundary, the reality will differ slightly from Eq. 3 for frequencies above 0.8THz due to the focal depth - frequencies below 0.8THz will still be in focus.

We can express the pulse resulting from the reflection at the air-quartz boundary, $E_1(\tau , \omega , t)$ in terms of $E_{in}(\tau , \omega , t)$:

4 $$\begin{aligned} \mathcal {F}\{E_1(\tau , t)\} = \mathcal {F}\{E_{in}(\tau , t)\}\frac{r_{aq}(\omega )}{T_1(x,y,\omega )}, \end{aligned}$$

where $r_{aq}(\omega )$ is the reflection coefficient at the air-quartz boundary.

The sample pulse $E_{sample}(\tau , \omega , t)$ is given by:

5 $$\begin{aligned} \mathcal {F}\{E_{sample}(\tau , t)\} = \mathcal {F}\{E_{in}(\tau , t)\}T_2(x,y,\omega )r_{sample}(\omega ), \end{aligned}$$

where $T_2(x,y,\omega ) = t_{qa}(\omega ) e^{-\alpha _q(\omega ) d_q(x,y)} e^{-j\frac{\omega }{c}n_q(\omega )d_q(x,y)}$, $t_{qa}(\omega )$ is the transmission coefficient at the quartz-air boundary, and $r_{sample}(\omega )$ is the reflection coefficient at the quartz-sample boundary. $T_2(x,y,\omega )$ corresponds to the transmission of the light from the quartz-to-sample boundary through to the air on the other side of the quartz. Note that ultimately it is the parameter $r_{sample}(\omega )$ that indicates the sample properties.

The first pulse $E_1(\tau , \omega , t)$ can be used to deconvolve the response function of the incident electric field $E_{in}(\tau , \omega , t)$ from the measured response of the sample $E_{sample}(\tau , \omega , t)$. We can calculate the corrected signal in the frequency domain:

6 $$\begin{aligned} M_{c}(x,y,\omega ) = \frac{\mathcal {F}\{E_{sample}(\tau , x,y,t)\}}{\mathcal {F}\{E_{1}(\tau ,x,y, t)\}} = Q(x,y,\omega )r_{sample}(\omega ) \end{aligned}$$

where

7 $$\begin{aligned} Q(x,y,\omega ) = \frac{T_2(x,y,\omega )T_1(x,y,\omega )}{r_{aq}(\omega )}. \end{aligned}$$

$Q(x,y,\omega )$ is independent of $\tau$ and the input electric field shape, consisting of constants that depend only on variations in the thickness of the quartz imaging window.

The change in the measured signal as a function of time after correction can be determined using:

8 $$\begin{aligned} \Delta M_{c}^{\tau }(\tau , \omega ) = \frac{M_{c}(\tau , \omega )}{M_{c}(90 \text { min}, \omega )}. \end{aligned}$$

The amplitude $|\Delta M_{c}^{\tau }(\tau , \omega )|$ and phase $\arg [\Delta M_{c}^{\tau }(\tau , \omega )]$ of the change in the corrected signal are shown in Fig. 7c and d for the 90-min measurement of air at a single point. The systematic change in phase as a function of $\tau$ is completely removed. However, the random noise in the measurement is increased.

The increase in random noise is due to the combination of noise from both the first and second pulses. This can be verified by measuring the ratio of the standard deviation of the measured amplitude of the 90-min air measurement at 0.5 THz before and after the correction:

$$\begin{aligned} ratio = \sigma (|\Delta M_{c}^{\tau }(\tau , \omega =0.5THz)|)/\sigma (|\Delta M^{\tau }(\tau , \omega =0.5THz)|) \approx \sqrt{2} \end{aligned}$$

The absolute increase in random noise is negligible in this application. Furthermore, the correction eliminates the need for deconvolution with a separate reference measurement, which is typically required in THz reflection spectroscopy [2, 12, 15, 17, 18].

**Fig. 9:**
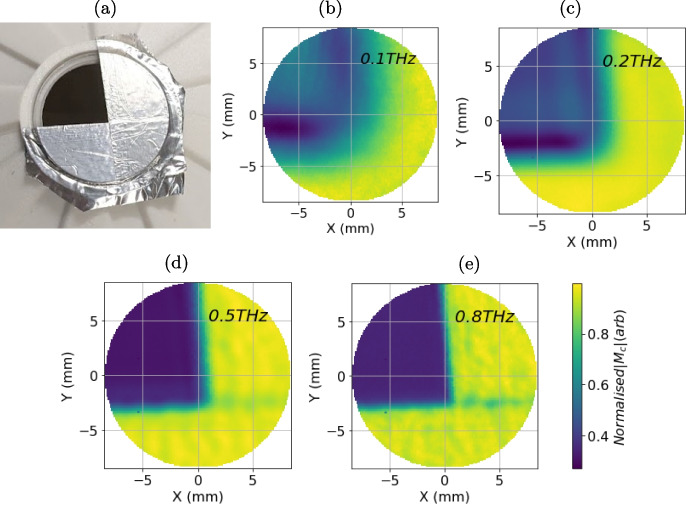
Images taken of the aluminium tape stuck to the quartz window of the PicoBot as shown in **a**. The images show the amplitude of the normalised corrected signal calculated using Eq. 6 at **b** 0.1THz, **c** 0.2THz, **d** 0.5THz, and **e** 0.8THz

This correction also significantly reduces noise associated with spatial variation in the measurement, as shown in Fig. 8a and b. The change in the measurement of air over the quartz window can be calculated using the corrected measurements:

9 $$\begin{aligned} \Delta M_{c}^{x,y}(\omega , x,y) = \frac{M_{c}(\omega , x,y)}{M_{c}(\omega , x=0,y=0)}. \end{aligned}$$

The amplitude $|\Delta M_{c}^{x,y}(0.5 \text {THz}, x,y)|$ and phase $\arg [\Delta M_{c}^{x,y}(0.5 \text {THz}, x,y)]$ at 0.5 THz are shown as a function of position in Fig. 8c and d, respectively. Compared to the images produced without the correction in Fig. 8a and b, there is a lower range of noise in both amplitude and phase. This is quantified by the standard deviation $\sigma$ given in each plot. The standard deviation for Fig. 8d (0.0036) is nearly an order of magnitude lower than for the uncorrected image in Fig. 8b (0.023).

Single-point measurement data do not improve as much as for the image data through this correction process. This is because, during the raster scan, the movement of the Menlo reflection head causes the attached fibre optic cables to move as well. This movement slightly alters the signal amplitude and phase during imaging, which does not apply to single-point measurements. In short, while the stability correction only improves point measurements slightly, it significantly reduces noise in images.

**Fig. 10:**
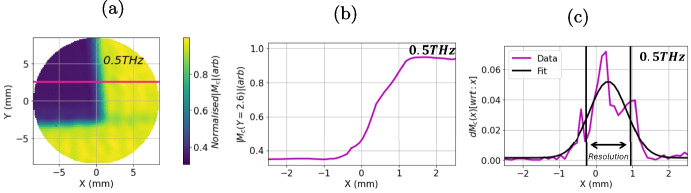
An example of the method used to calculate the resolution in the *X* direction. **a** An image of tape at 0.5THz with a black line indicating the profile used to find the *X* resolution at 0.5THz. **b** The amplitude at 0.55THz as a function of *X* along the black line. This can then be differentiated and fit with a Gaussian peak **c** the width of the fitted Gaussian can then be used to find the resolution of the image at 0.5THz in the *X* direction

## Spatial Resolution

The spatial resolution of the PicoBot is determined by the following test measurements using highly reflective aluminium tape. The aluminium tape is attached to the PicoBot imaging window, as illustrated in Fig. 9a. During imaging, the tape should appear as a sharp edge. The apparent width of this edge is indicative of the resolution.

The resolution of the PicoBot imaging system is limited by the diameter of the diffraction-limited Airy disk of the THz spot on the quartz window and is tuned by setting the value of *dX* for the raster scan path (indicated in Fig. 3b). The diffraction limit decreases with frequency from an upper limit of approximately 5 mm, meaning higher frequencies result in better resolution. Figure 9 shows images of the tape on the quartz window by plotting $|M_{c} (x,y,\omega )|$ at frequencies of 0.1, 0.2, 0.5, and 0.8THz. The line at the boundaries of the tape clearly sharpens at higher frequencies.

As illustrated in Fig. 3b, the scan path is not symmetrical in the *X* and *Y* directions. The number of pixels measured along each line is approximately 100, while the number of lines depends on the *dX* setting. This means that the *X* and *Y* resolutions of the PicoBot need to be measured separately.

The amplitude of an image at a fixed *y* coordinate can be used to find the *x* resolution, and vice versa. Figure 10a shows an amplitude image for $\omega = 0.5THz$. Figure 10b shows the amplitude as a function of *X* for fixed *Y* = 2.6mm. This resembles a broadened step function. The differential of this line,

10 $$\begin{aligned} dM_c(x)[\text {wrt}:x] = \frac{d|M_{c}(\omega =0.55, x, y=2.6)|}{dx} \end{aligned}$$

gives a Gaussian peak, the width of which can be taken as the resolution. An example is shown in Fig. 10c.

**Fig. 11:**
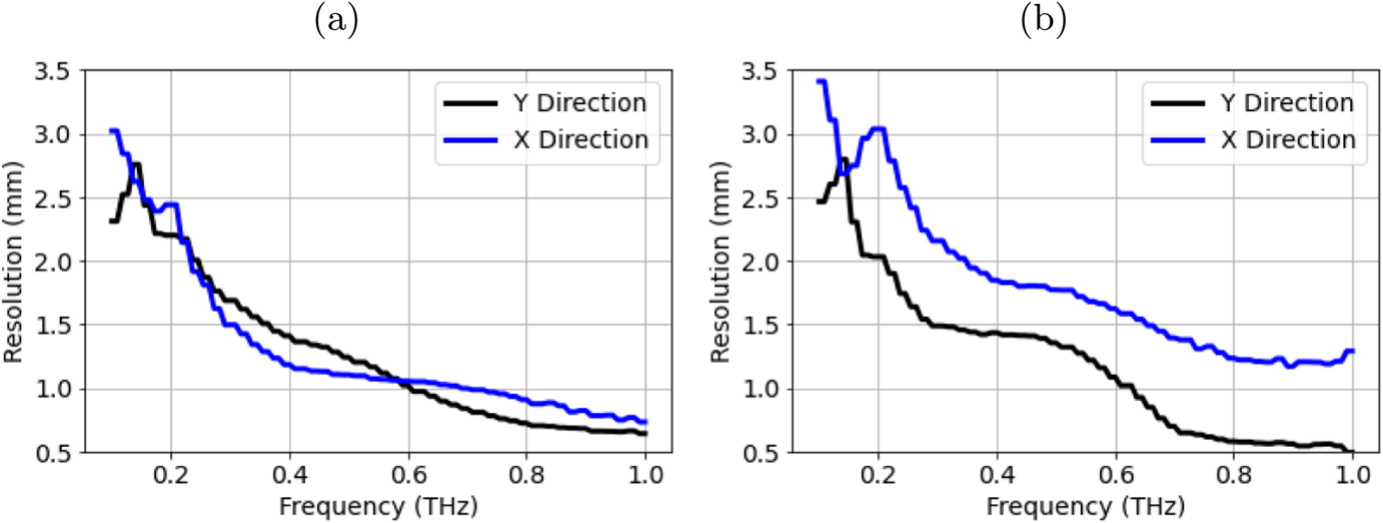
Resolution as a function of frequency in the *X* and *Y* direction **a** when the PicoBot setting dX=0.1mm is used and **b** when dX=1 mm is used, this setting is used during in vivo measurements

The resolution has been calculated for $dX = 0.1mm$ and $dX = 1.0mm$, and is shown between 0.1 and 1THz in Fig. 11a and b, respectively. $dX=0.1mm$ was chosen because it is an order of magnitude smaller than the diffraction-limited spot size of the THz beam, and with this setting, the resolution varies from 3 mm at 0.1THz to 0.8mm at 1.0THz. When $dX = 1.0mm$, the resolution in the *y* direction remains similar, but the *x* resolution is poorer, ranging from 3 mm at 0.2THz to 1.2mm at 1.0THz. Resolution requirements vary depending on application: cancer margins are measured to within 2 mm in the UK. The PicoBot is therefore set to $dX=1.0mm$ for patient measurements to reduce image acquisition time while still providing a suitable resolution (1.6mm in the *y* direction and 1 mm in the *x* direction at 0.6THz).

**Fig. 12:**
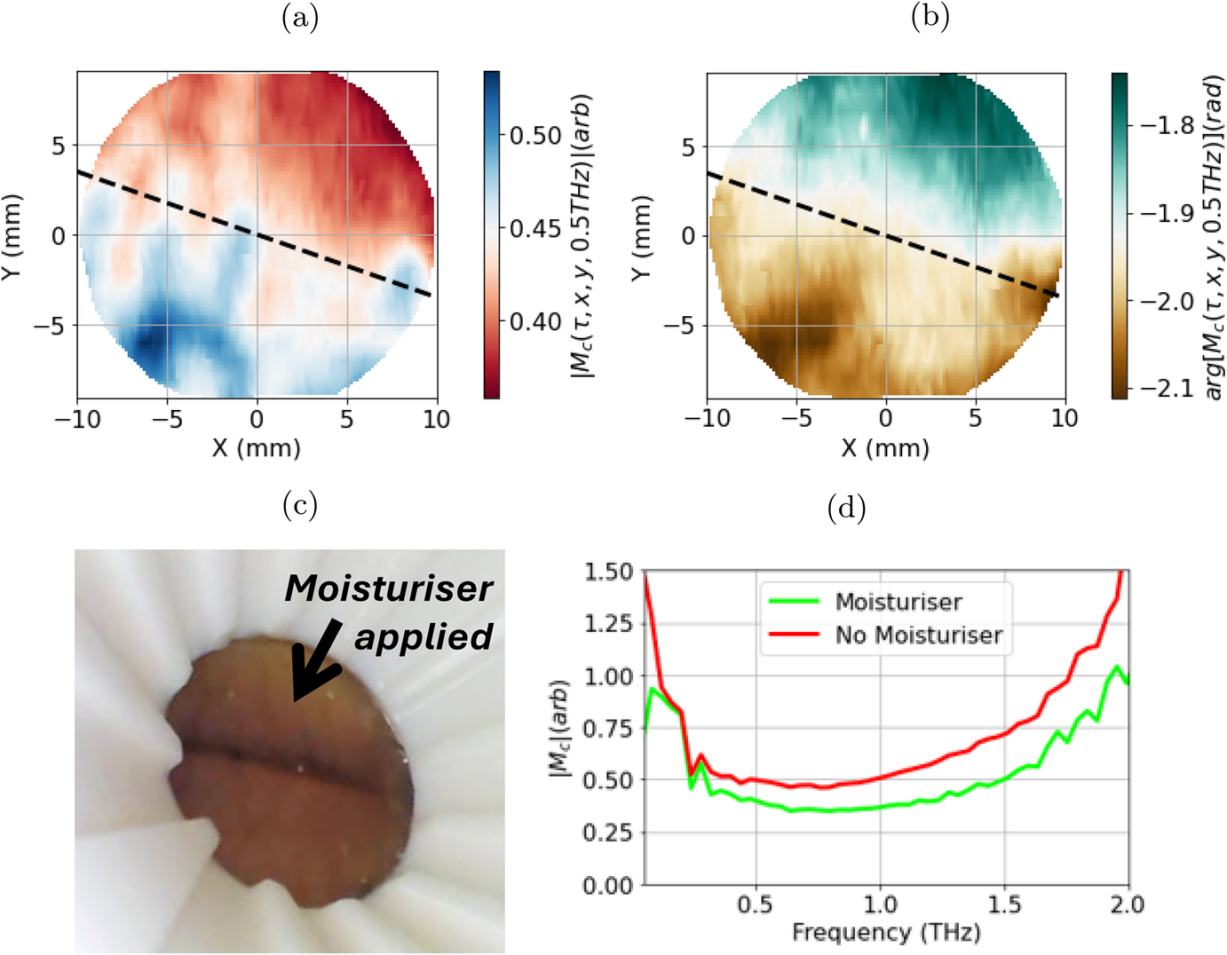
The THz image showing the amplitude of the corrected signal $|M_c|$. The THz image showing the phase of the corrected signal $arg[M_c]$. In both images a dashed line indicates the boundary at which moisturiser was applied. A photo of the region being measured taken using an endoscopic camera mounted inside the PicoBot, moisturiser has only been applied on one side of the line as indicated. $|M_c|$ for an unmoisturised region and a moisturised region

## In Vivo Image Example

As an example, the PicoBot has been used to take an in vivo image of a person’s forearm, using the protocol outlined in Fig. 4. The photo in Fig. 12c shows the region of the forearm that was measured, taken using an endoscopic camera inside the PicoBot probe. To demonstrate the sensitivity of THz radiation to skin hydration, a moisturiser (Liz Earle Skin Repair) was applied on the side of the line indicated in the photo 10 min prior to the THz measurement, and the excess was wiped away before the measurement. This gave time for the moisturiser to be absorbed and it was not visible on the skin. Figure 12a and b show the amplitude and phase of the corrected measurement $M_{c} (x,y,\omega = 0.5THz)$ (calculated using Eq. 6). A clear distinction can be seen between the region where the moisturiser was applied and the rest of the skin, with a smaller $|M_{c} (x,y,\omega = 0.5THz)|$ values, and higher $arg[M_{c} (x,y,\omega = 0.5THz)]$ values where the moisturiser was applied. This is consistent with previous point measurement studies conducted by Lindley-Hatcher et al. [7]. The boundary between the moisturised and unmoisturised skin is not as sharp as in the aluminium tape experiment as the moisturiser will spread out within the skin from where it is applied. Figure 12d shows the amplitude of the corrected signal spectrum $|M_{c} (x,y,\omega = 0.5THz)|$ for both the moisturised and unmoisturised regions of the image. It can be seen that the unmoisturised region has a larger signal across the entire spectrum, with the contrast increasing at high frequencies.

## Discussion

We have shown how THz instrumentation can be integrated to acquire in vivo measurements of people in a repeatable and accurate way. The PicoBot probe is physically robust, with all the optics enclosed and protected in a wipe-clean casing. The time taken for the image measurement is currently 4 min and we are continually working to reduce this.

The resolution is limited by the diffraction-limited spot size of the THz beam. In Fig. 11b, the *y* resolution is generally better than the *x* resolution when $dX = 1mm$. This compromise in resolution has the benefit of reducing the THz imaging time. However, imaging speed is limited not by the THz pulse acquisition rate of 10 Hz, but by the *XY* stage speed of 2.3mm/s. A faster stage could be employed; for example, a 10Hz acquisition rate with a limiting resolution of 1 mm could increase scanning speed to 10 mm/s. Using the same $dX = 1mm$ and the same scanning path shown in Fig. 3b, the image could be acquired in just 90 s. A galvo scanner could also be employed to speed up the scanning rate, however, this would require the optics and the casing of the PicoBot to be redesigned, and care would need to be taken to ensure reliable performance. After improving the scanning speed, the limiting factor in measurement speed becomes the THz pulse acquisition rate. This can be improved by using faster commercially available mechanical stages like those used in this study or an ASOPS, which can measure THz pulses at 100Hz. Further work is being carried out to improve imaging speed without compromising resolution. Current NHS clinical guidelines require a 2 mm margin around the imaged cancer to be removed [19], so further improvements to resolution are not required for this application.

The THz images in Fig. 12 show that different levels of hydration in the skin result in contrast across an image. This is a promising sign that skin cancer could be evaluated using this device as the vascularity of cancer can manifest as increased hydration [2]. Skin conditions that affect skin hydration, such as eczema and psoriasis [12], could also be evaluated. Noteworthy, in this example, only the amplitude and phase at one frequency value are plotted; however, the PicoBot records over a bandwidth of approximately 0.05THz to 1.0THz. This means that there is much more information available in the data than shown in these example images. More work is needed to fully understand the clinical value of all the information.

## Conclusion

We have developed a THz imaging device for in vivo imaging and evaluation of skin. The PicoBot is capable of taking 20 by 100-pixel images with 1 mm resolution at 0.5THz in 4 min, by using a continuous raster scanning method. The resolution is limited by the diffraction-limited spot size of the THz beam. The PicoBot is positioned using a robotic arm, controlled by a pressure sensor to enable consistent and repeatable measurements. A faster moving *XY* stage would allow imaging speeds of 80 s without compromising imaging and work is being conducted to implement this.

The PicoBot is currently being utilised in the TeraBotics study to image skin cancer patients at the University Hospitals of Coventry and Warwickshire Trust. The data from this study will aid in finding diagnostic criteria for skin cancer using THz radiation and pave the way for further development into a viable clinical evaluation tool for skin conditions.

## Data Availability

All the data used to produce the plots in this paper are available on Figshare: https://doi.org/10.6084/m9.figshare.28588589
